# Acute and chronic immunomodulatory response mechanisms against *Toxocara canis* larvae infection in mice

**DOI:** 10.1590/S1984-29612022056

**Published:** 2022-11-07

**Authors:** Jéssica Lopes Borchard, Neida Lucia Conrad, Natália Berne Pinto, Micaele Quintana de Moura, Maria Elisabeth Aires Berne, Fábio Pereira Leivas Leite

**Affiliations:** 1 Programa de Pós-graduação em Microbiologia e Parasitologia, Instituto de Biologia, Universidade Federal de Pelotas – UFPel, Pelotas, RS, Brasil; 2 Biotecnologia, Centro de Desenvolvimento Tecnológico, Universidade Federal de Pelotas – UFPel, Pelotas, RS, Brasil

**Keywords:** Immune response, toxocariasis, cytokines, Ym1, Resposta imune, toxocariasis, citocinas, Ym1

## Abstract

The objective of this work was to evaluate the early and late immunological modulation of an experimental infection of *T. canis* larvae in mice. Mice were infected with 100 infective larvae and euthanized at different period: 24, 48 hours post infection (HPI), 15- and 30 days post infection (DPI). The humoral response was evaluated by indirect ELISA. Quantitative RT–PCR (qPCR) was used to quantify the mRNA transcription of cytokines *IL4*, *IL10*, *IL12* and *Ym1* in the early and late infection periods. Infection with *T. canis* was able to generate specific total IgG at 15- and 30- DPI. Analyzing the IgG isotype revealed a significant differentiation for IgG1 compared with IgG2a, IgG2b and IgG3, characterizing a Th-2 response. Evaluating the gene transcription at the early phase of infection, higher transcription levels of *IL10*, *IL4* and *Ym1* and a downregulation of *IL12* were observed. By the late phase, increased transcription levels of *IL4*, *Ym1* and *IL12* were observed, and downregulation of *IL-10* transcription was observed. The data obtained suggest that during experimental infection with *T. canis*, the participation of the IL4, IL10, IL12 cytokines and Ym1 can play an important role in *T. canis* immunomodulation.

## Resumo

O objetivo deste trabalho foi avaliar a modulação imunológica precoce e tardia da infecção experimental de larvas de *T. canis* em camundongos. Estes foram infectados com 100 larvas infectantes e eutanasiados em diferentes períodos: 24 e 48 horas pós infecção (HPI), 15 e 30 dias após a infecção (DPI). A resposta humoral foi avaliada por ELISA indireto. RT-PCR quantitativo (qPCR) foi usado para quantificar a transcrição de mRNA das citocinas *IL4*, *IL10*, *IL12* e *Ym1* nos períodos de infecção precoce e tardia. A infecção por *T. canis* foi capaz de gerar IgG total específico aos 15 e 30 DPI. A análise do isótipo IgG revelou uma diferenciação significativa para IgG1 em comparação com IgG2a, IgG2b e IgG3, caracterizando uma resposta Th-2. Avaliando-se a transcrição gênica na fase inicial da infecção, foram observados níveis mais elevados de transcrição de *IL10*, *IL4* e *Ym1* e a regulação negativa de *IL12*. Na fase tardia, foram observados níveis aumentados de transcrição de *IL4*, *Ym1* e *IL12*, e foi observada regulação negativa da transcrição de IL-10. Os dados obtidos sugerem, que durante a infecção experimental com *T. canis*, a participação das citocinas IL4, IL10, IL12 e Ym1 podem desempenhar um papel importante na imunomodulação de *T. canis*.

## Introduction

Toxocariasis is a neglected zoonosis caused by helminth larvae of the species *T. canis* and *T. cati,* with most cases associated with *T. canis.* The disease may lead to diverse syndromes in humans, such as visceral and ocular toxocariasis (Othman et al., 2013). Human toxocariasis occurs through the ingestion of embryonic eggs of *T. canis* containing L3 larvae, through the consumption of water and food contaminated by eggs (Antunes et al., 2013), direct contact with infected dogs and cats (Romero Núñez et al., 2013), and consumption of undercooked meat (Overgaauw & van Knapen, 2013). Humans are considered accidental hosts; in these, after infection, the larvae do not evolve to their adult stage, and there is also no migration of this to the small intestine, as in definitive hosts. However, the larvae remain viable, encapsulated for months or years (Despommier, 2003; Rubinsky-Elefant et al., 2010) and can reach several organs, such as the lungs and liver (Chen et al., 2012; Smith et al., 2009).

The immune defense against helminth infections is mediated by the activation of Th2 cells, eosinophils and mast cells (Allen & Maizels, 2011; Maizels et al., 2018). Regardless of the extensive description of helminths immune toward a Th-2 response, the mechanism(s) responsible are not totally understood.

Cytokines play an important role in the modulation of the type of immune response (e.g., Th-1, Th-2, Th-17 or Treg) (Kaufmann, 2007). The presence of IL4 in the early stages of T cell priming induces an immune response toward Th-2 (Noben-Trauth et al., 2000, 2002). However, the origin of IL4 is still a matter of discussion and is ascribed to different immune cell populations. The production of IL10 by macrophages and DCs may also play a role in directing a polarizing Th2-type response by suppressing a Th1-type response (Oleszycka et al., 2018). During larval invasion, tissue damage stimulates the release of alarmins. These alarmins stimulate Th-2 cytokines (e.g., IL4) by innate cells (e.g., ILCs) and CD4+ Th-2 lymphocytes. This Th-2 inflammation is believed to be important in the activation of alternative activated macrophages, such as M2 macrophages, that modulate the immune response toward Th2 cells (Edwards et al., 2006; Gordon, 2003). A protein that has been considered a marker of M2 macrophages is protein Ym1, which is constantly transcribed in Th2 immune responses (Faz-López et al., 2019).

Understanding the immunological changes that occur in the organism during invasion by *T. canis* is extremely important, since understanding how this helminth modulates immunity will allow the development of immunological protection against toxocariasis (Maizels, 2013). The aim of the present study was to evaluate immune modulation mechanisms mediated by *T. canis* in experimentally infected mice during early and late periods of infection.

## Material and Methods

### Experimental design

In the present study, 50 female Balb/c mice were randomly separated in 5 groups of 10 mice each (Crawley, 2002). The mice were kept in a controlled environment (22 ± 2 °C) with *ad libitum* access to feed and water.

The experimental infection was performed by the oral administration of 100 larvae (L3) of *T. canis* per mouse. The method of infection was through the intragastric tube (Avila et al., 2012). In this work, five groups containing 10 mice each were infected with 100 infective larvae (L3). The experimental groups were euthanized at different periods: Group 1. 24 hours post infection (HPI), Group 2. 48 HPI, Group 3. 15 days post infection (DPI) and Group 4 on the 30 PDI. Group 5 was the control group, which was not infected.

The total specific anti-TES IgG and its immunoglobulin isotypes were evaluated by analyzing the sera of the mice after blood collection using the retro-orbital plexus. The gene transcription of cytokines *IL4, IL10, IL12* and *Ym1* protein was also evaluated in splenocytes after experimental infection with 100 larvae for 24, 48 HPI, 15 and 30 DPI. The recovery of *T. canis* larvae was performed at 24 and 48 DPI using the liver tissue digestion technique (Xi & Jin, 1998).

### Obtaining *T. canis* larvae

*T. canis* eggs were collected and incubated according to Avila et al. (2012), and the larvae were extracted by physicochemical methods following Savigny (1975) and maintained in culture as proposed by Falcone et al. (2001) and Perkins (1992). Larval viability was confirmed by microscopic examination of motility.

### Obtaining the excretion and secretion antigen from *T. canis* (TES)

The excretory-secretory material of *T. canis* larvae (TES) was obtained according to Savigny (1975) and Savigny et al. (1979), with some modifications. The larvae were grown in RPMI 1640 medium (Cultilab, Campinas, Brazil), with medium changes being made during their cultivation period (supplemented with 25 mM HEPES, 1% glucose, 100 μL/mL penicillin, 100 μg/mL streptomycin, 0.4 μg/mL ofloxacin and 50 μg/mL fungizone). The supernatant was collected, and a 200 mM protease inhibitor (phenyl-methyl-sulfonyl-fluoride) was added (5 µl/ml of collected medium) after the supernatants were frozen at -20 °C in sterile tubes. After the supernatant was filtered through a 0.22 µ Millipore membrane and in sequence through a 10 Millipore membrane. Dialysis was performed in distilled water and centrifuged at 4 °C at 12,000 × *g* for 60 minutes. The antigen underwent the lyophilization process, and for protein quantification, the antigen was dissolved in free water. The antigen concentration was measured using a BCA kit (Pierce, Rockford, IL, USA).

### Evaluation of the humoral immune response of experimental animals

The assessment of total serum IgG levels (anti-*Toxocara*) was performed by indirect immunoenzymatic assay (ELISA) using TES as the antigen at a concentration of 1 μg/ml and serum dilution of the primary antibody (1:50), as described by Avila et al. (2012). For the secondary serum, horseradish peroxidase (HRP)-conjugated anti-mouse IgG antibody produced in goat (Sigma-Aldrich, St. Louis, Missouri, USA), a 1:5000 dilution in PBS-T, was used. Serum samples were examined individually and in duplicate, and readings were performed at a wavelength of 492 nm. The evaluated isotypes were IgG1, IgG2a, IgG2b and IgG3, following the recommendations of Monoclonal Mouse Antibody Isotyping Reagents (Sigma-Aldrich, St. Louis, Missouri, USA). The concentration of antigen used in sensitization and the dilution of the primary serum were the same as those used for the evaluation of total IgG. Each sample (pool of sera) was examined in triplicate to obtain the mean absorbance. The secondary antibodies anti-mouse IgG1, IgG2a, IgG2b and IgG3 (Sigma-Aldrich, St. Louis, Missouri, USA) produced in goat were diluted 1:2000 in PBS-T and applied to the plates. After, the peroxidase-conjugated anti-goat antibody produced in rabbit (Sigma-Aldrich, St. Louis, Missouri, USA) was diluted 1:4000 in PBS-T was added.

### Cultivation of splenocytes and quantitative polymerase chain reaction (qPCR)

The cultivation of splenocytes was carried out from all mice of each group. The spleens were removed and macerated, and the obtained splenocytes were suspended in a balanced HANK'S solution (without Ca and Mg). Afterward, the cells were centrifuged, and the pellet was suspended in lysis solutions (0.8% ammonium chloride), followed by washing and suspension in RPMI 1640 (Cultilab, Campinas, Brazil) with 10% fetal bovine serum (SFB) (Cultilab, Campinas, Brazil). Cells were counted in a Neubauer chamber, and 2 x 10^6^ cells were cultured per well in 24-well plates and incubated for 24 hours in an atmosphere of 5% CO_2_ at 37 °C. After this period, the cells were stimulated with RPMI + fetal bovine serum (10%), Concavalin A (5 µg/ml) or TES (10 µg/ml). After cultivation, the cells were collected in TRIzol (Sigma-Aldrich, St. Louis, Missouri, USA) and stored at -70 °C. RNA extraction was performed using the TRIzol method and cDNA synthesis from 400 ng/μl of mRNA, according to the manufacturer's instructions (Applied Biosystems, Foster City, CA, USA). For the quantitative method of polymerase chain reaction (qPCR), it was used for the amplification of segments of the *IL12* genes (F: AGCACCAGCTTCTTCATCAGG, R: CCTTTCTGGTTACACCCCTCC), *IL4* (F: CCAAGGTGCTTCGCATATTT, R: ATCGAAGAGTAGGAGT, R: TTTGTCCTTAGGAGGGCTTCCTCG), *IL10* (F: TTTGAATTCCCTGGGTGAGAA, R: ACAGGGGAGAAATCGATGACA) *Ym1* (F: CACAGGTCTGGCAATTCTTCTG, R: TTTGTCCTTAGGAGGGCTTCCTCG), and β-actin (F: AACGCCCTTCATTGAC, R: TCCACGACATACTCAGCAC). qPCR was performed with 1 μL of cDNA, 5.0 μL of SYBR Green (Invitrogen, Carlsbad, CA, USA), 0.25 μM of each initiator oligomer and 3.5 μL of RNase-free water (Gibco, Gaithersburg, MD, USA) in a total volume of 10 μL. The temperatures used were as follows: denaturation at 95 °C for 5 min, followed by 40 cycles with denaturation at 95 °C for 30 s, annealing at 65 °C for 60 s and extension at 72 °C for 60 s and final extension at 72 °C for 5 min. All samples were analyzed in triplicate. From the values of cycle threshold (Ct) obtained, the variation in gene transcription was calculated by comparison with the expression of β-actin (control). Method 2- (^ΔΔ^Ct) was used for the relative quantification of gene transcription between samples (Livak & Schmittgen, 2001).

### Statistical analysis

Data analysis was performed using Past 1.0.0 software. The Shapiro–Wilk test was used to verify the normality of the data. Data that did not show a normal distribution were transformed into a base 10 logarithm. After two-way analysis of variance (ANOVA), the data underwent a Tukey test to compare the means. Significance was set at p <0.05. The graphics were generated using the GraphPad Prism 7.0 program (GraphPad Software Inc; San Diego, CA, USA).

## Results

### Infection

*T. canis* infection was confirmed by the mean of larval recovered at 24 and 48 HPI from the mice livers. Was observed a mean recovered of 9.5 larvae (± 3.35 Standard Deviation) at 24 HPI and 13.3 (± 7.15 SD) at 48 HPI.

### Dynamics of serum total anti-TES IgG and isotypes

**Figure 1:**
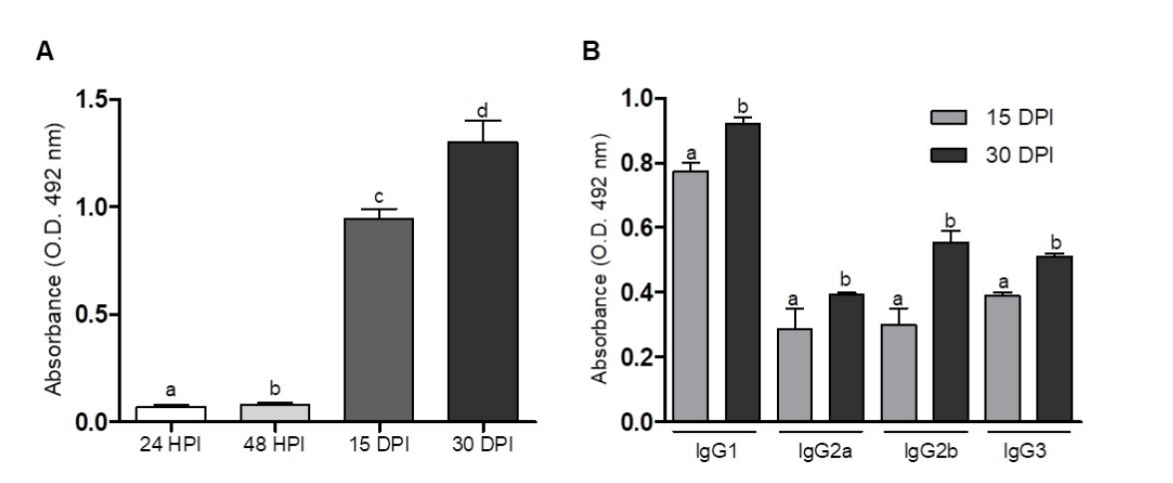
Analysis of serum IgG dynamics. (A) Total specific anti-TES IgG levels determined by indirect ELISA of mice infected with *T. canis* larvae. The data represent the mean ± standard error of absorbance; (B) IgG isotype profile determined by indirect ELISA of mice infected with *T. canis* larvae. The statistical analysis was performed by two-way ANOVA followed by Tukey’s multiple comparisons test. HPI, hours post infection and DPI, days post infection.

Total serum IgG specific for TES was significantly higher (p<0.05) in the infected mouse group at 15- and 30-DPI than in the control group (uninfected). A significantly higher IgG response was also observed at 30 DPI than at 15 DPI (p<0.05) (Figure 1A). Evaluating the IgG isotypes, we observed that all studied isotypes had a similar IgG pattern at 15- and 30-DPI. It is worth noting that the IgG2b isotype at 30 DPI showed an ~2-fold increase in its level compared with that at 15 DPI (Figure 1B).

### Cytokine transcription

**Figure 2:**
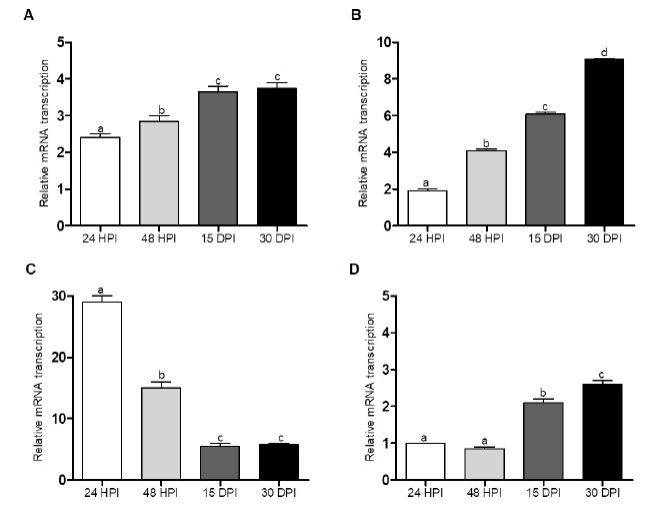
qPCR mRNA transcription for IL4 (A), Ym1 (B), IL10 (C) and IL12 (D). The data represent the mean (±S.E.M) of mRNA transcription in splenocytes from mice infected with *T. canis* larvae. The splenocytes were stimulated in vitro with TES at the different time points evaluated (HPI, hours post infection and DPI, days post infection). The relative cytokine mRNA transcription was determined by the 2-ΔΔCT method. The statistical analysis was performed using one-way ANOVA followed by Dunnett’s test. Similar letters indicate no statistical difference (p ˃ 0.05), while different letters indicate a statistical difference (p < 0.05) between the experimental groups.

The splenocyte mRNA transcription of *IL4* and *Ym1* showed a similar pattern but with different transcription levels at all points studied. *IL4* had a significant (p<0.05) increase in mRNA transcription at 24 and 48 HPI and by 15 DPI, maintaining the transcription levels until 30 DPI (Figure 2A). For *Ym1*, transcription was significantly elevated at 24 hours and continued to increase up to 30 DPI (Figure 2D). *IL10* transcription was significantly higher at 24 HPI, reducing its transcription at 48 HPI and by 15 DPI, keeping the transcription levels until 30 DPI (Figure 2B). Transcription of *IL12* was not observed at 24 and 48 HPI, was only observed at low levels by 15 DPI and showed a significant (p<0.05) increased (~3-fold) transcription by 30 DPI (Figure 2C).

## Discussion

In this study, we evaluated the Th2 immunomodulation response mechanism of Balb/c mice experimentally infected with *T. canis* larvae. *T. canis* infection is associated with a variety of immunomodulatory mechanisms allowing infection and evasion of the immune system and, by doing so, persistence in the host. A humoral immune response with elevated IgG1 isotype and cytokine transcription characterizing a modulation toward the Th2 response by *T. canis* larvae was observed.

Evaluating the specific anti-TES IgG, we observed an increase in the IgG levels from 15 to 30 DPI, suggesting that the *T. canis* larvae modulated a specific humoral immune response against TES. Assessing the IgG isotypes, it was observed that by 15 DPI, the mice had an immune response characterized by the presence of IgG1 significantly higher than the other isotypes (IgG2a, IgG2b, and IgG3), maintaining up to the 30 DPI. This finding corroborated other studies where the isotype with higher levels was IgG1 (Gazzinelli-Guimaraes & Nutman, 2018; Muallem & Hunter, 2014), with IgG1 considered the main IgG present in *T. canis* infection (Novák et al., 2017). The IgG subclasses induced after infection are an indirect indicator of the relative contribution of Th2/Th1-type responses. Analyzing the IgG2a, IgG2b and IgG3/IgG1 ratios at the 15 and 30 DPI, we observed a relation with the Th2 response. This observation might be important since the *in vivo* outcome of the IgG-mediated immune response is largely determined by the balance of activating or inhibitory signals transduced by the FcγRs (Nimmerjahn & Ravetch, 2006). IgG1 has high affinity to engage inhibitory FcγRIIb receptors, whereas the IgG2a isotype has greater affinity for activating FcγRIV and, in doing so, gives IgG1 antibodies minimal activation activity (Nimmerjahn & Ravetch, 2005). The levels of IgG2b are worth noting, and IgG2b was the isotype that had the highest response by 30 DPI, increasing ~2.5-fold compared to 15 DPI (Figure 1B). One may speculate that by the 30 DPI, a modulation toward a mixed Th2/Th1 response may be occurring for infection resolution. Experiments carried out with Balb/c mice infected with *T. canis* revealed a mixed immune response with no predominance of Th1 or Th2 lymphocytes (Hamilton et al., 2008). This modulation might be supported by the increased level of *IL12* transcription, steady *IL4* transcription levels, and reduction of *IL10* transcription observed on Day 30 (Figure 2). However, the meaning of the IgG2b observation was out of the present study scope.

The production of cytokines in splenocytes from *T. canis*-infected mice is characterized by Th2 cytokines such as IL4 (Faz-López et al., 2013), the regulatory cytokine IL10 (Malheiro et al., 2008) and the inhibition of proinflammatory INF-y and IL12 cytokines (Długosz et al., 2015; Kuroda et al., 2001). It is well known that *T. canis* infection in Balb/c induces the expression of IL4, IL5 and IL10, and the expression of these cytokines may play a role in suppressing Th1 lymphocytes (Frantz et al., 2010; Hamilton et al., 2008). IL4 expression is a key player in parasite infection in both mice and humans (Brys et al., 2005), playing an important role in B cell class switching and cellular migration into peripheral tissue (van Panhuys et al., 2011). In the present study, significant splenocyte *IL4* transcription was observed as early as 24 hours up to 30 days after *T. canis* larval infection. This finding is quite interesting since an innate IL4 response at the spleen level is modulated by *T. canis* larvae in the first hours of infection and was maintained until the end of the experiment. IL4 promotes the B cell response by inducing the exchange of IgG isotype to IgG1 (Finkelman et al., 1990; Zhu et al., 2010), suggesting that modulation of *IL4* might lead to the exchange of IgG isotype to IgG1 observed in this study (Finkelman et al., 1990; Zhu et al., 2010).

IL-10 is a cytokine produced mainly by CD4^+^ Foxp3 regulatory T lymphocytes, and its biological activity depends on its concentration, which may cause inhibition or stimulation of immune cells (Gasbarre, 1997; Oleszycka et al., 2018). The activation of TLR receptors and the MyD88 transcription factor has been related to the activation of immune cells, which are secretors of IL10 (Hayashi et al., 2013). According to our results, high *IL10* transcription was observed in mice infected with *T. canis* after 24 and 48 HPI, a prompt innate response that may play a role by antagonizing the *IL12* transcription observed. Notably, *IL10* transcription was reduced by 15- and 30-DPI, suggesting a role in the evolution/resolution of *T. canis* infection (Othman et al., 2011).

There are different cell populations that are responsible for IL12 production, such as dendritic cells (DCs), monocytes, macrophages, and neutrophils (Bliss et al., 2001; Ferragine et al., 2013; Teixeira et al., 2010). In the present study, we observed suppression of *IL12* transcription. This finding was expected, since it is well established that *T. canis* infection induces IL12 suppression, and this effect contributes to the *T. canis* modulation of the immune response toward a Th2 (Avila et al., 2016; Kuroda et al., 2001). Notably, by the late infection period (15- and 30-days PI), an increase in *IL12* transcription was observed. Cheng et al. (2016) reported that increased IL12 played a role in *Schistosoma japonicum* reduction; similarly, Pilarczyk et al. (2008) demonstrated that IL12 reduced the *T. canis* parasitic load. Therefore, one may suggest that this increase in IL12 might be related to *T. canis* infection resolution.

During helminth infection, alternative activated macrophages (AAMacs) are key to promoting the Th2 response and are present at the infection site in the first 24 h after infection (Donnelly et al., 2005; MacDonald et al., 2002). AAMcs (also known as M2 macrophages) are classified phenotypically as macrophages induced by IL4/IL13 and have the markers Arg-1, mannose receptor, Fizz1 and Ym1 (Edwards et al., 2006; Gordon, 2003; Raes et al., 2002). The chitinase-like Ym1 protein is the most abundantly expressed gene in helminth-activated macrophages (Loke et al., 2002) and may be activated by helminth infection independently of IL4/IL13 signaling. Donnelly et al. (2008) identified that the antioxidant PrX induces Ym1 expression by macrophages and using anti-PrX neutralizing antibodies inhibited Ym1 expression as well as T cell differentiation toward a Th2 phenotype. Thus, its induction is critical for the development of the Th2 response (Brys et al., 2005; Donnelly et al., 2008; Falcone et al., 2001). Different authors have reported the presence of Ym1 in parasite infections such as *Brugia malay* (Falcone et al., 2001; Loke et al., 2002). *Litomosoides sigmodontis*, *Nippostrongylus brasiliensis* (Nair et al., 2005) and *Trichinella spiralis* (Chang et al., 2001). Recently, Faz-López et al. (2019) reported that experimental mice infected with *T. canis* larvae have an upregulation of Ym1, which favors parasite resistance. In the present study, increased *Ym1* mRNA transcription was observed in all analyzed periods. Notably, *Ym1* transcription increased from 24 hours to 30 DPI. Based on these observations, it is believed that Ym1 plays an important role in type 2 (Th2) modulation mediated by *T. canis*.

## Conclusion

The results from the present study demonstrated that *T. canis* larvae were able to modulate an immune response as early as 24 h until 30 days after infection. The innate and acquired immune responses were characterized by patterns of IgG isotypes, *IL4*, *IL10* and *IL12* cytokines and *Ym1* mRNA transcription. This finding paves the way to better understand the immune mechanism mediated by *T. canis* infection.
